# Do German Children Differ? A Validation of Conners Early Childhood™

**DOI:** 10.1177/1087054720907955

**Published:** 2020-03-14

**Authors:** Selina Türk, Simon Harbarth, Sebastian Bergold, Ricarda Steinmayr, Eva Neidhardt, Inge Kamp-Becker, Monika Equit, Katja Wunsch, Hanna Christiansen

**Affiliations:** 1 Philipps University Marburg, Germany; 2 TU Dortmund University, Germany; 3 University of Koblenz and Landau, Germany; 4 University of Saarland, Saarbrücken, Germany; 5 University of Rostock, Germany

**Keywords:** attention deficit/hyperactivity disorder, preschool, early childhood, development, parent, teacher, ratings

## Abstract

**Objective:** The present study aimed to validate the German version of the Conners Early Childhood (EC)™ among German-speaking children. **Method:** A total of 720 parental and 599 childcare provider ratings of 2- to 6-year-old children were surveyed throughout Germany. Validity was assessed by calculating exploratory factor analyses (EFAs) and confirmatory factor analyses (CFAs), and a series of multivariate analyses of variance (MANOVAs) to analyze associations between Conners EC™ symptom ratings and sociodemographic variables. In addition, parent and childcare provider ratings of Conners EC™ scales were correlated with a number of other well-validated German measures assessing preschoolers’ behaviors. **Results:** Although the EFA yielded different factors than the original scales, CFA revealed acceptable to good model fits. **Conclusion:** Overall, we confirmed the factor structure of the Conners EC’s™ American original within the German validation. The use of the American factor structure is justified and can be recommended to facilitate international research on psychopathology in early childhood.

Almost every fourth child or adolescent suffers from at least one mental health problem: The worldwide prevalence rate of mental disorders in children and adolescents is as high as 20% (Kieling et al., 2011; Polanczyk et al., 2015). Whereas the prevalence of mental disorders in school-age children and adolescents is well known and seems to be as high as in adults, with rates into adulthood persisting in as many as 50% (Patton et al., 2014), significantly less is known about the prevalence of mental disorders in *pre*school children. Research in this decade has therefore concentrated on the early development of mental health in preschool children (up to 6 years of age). Those studies documented that the prevalence of mental disorders in preschool children is already as high as in later years. For instance, a review by Egger and Angold (2006) reported a prevalence rate ranging from 14% to 26.4% of Diagnostic and Statistical Manual of Mental Disorders (*DSM*)-based mental health problems among preschoolers, which is comparable to the rates in later childhood and adolescence and also replicated in studies relying on the International Classification of Diseases (ICD; Skovgaard, 2010; see also Lavigne et al., 2009; Wichstrøm et al., 2012). Notably, mental disorders also appear stable from preschool to school age and can accompany severe functional impairments, such as underachievement at school (Spira & Fischel, 2005), peer rejection, and deficits in social functioning (Hoza et al., 2005; Murray-Close et al., 2010); they also function as a risk factor for psychopathology in later development (Angold & Egger, 2007; Bufferd et al., 2012; Bunte et al., 2014; Costello et al., 2003; Lahey et al., 2016). Taken together, an early manifestation of symptoms is associated with high rates of persistence, impairment, and comorbidity, especially if the symptoms remain unattended (S. B. Campbell, 1995; Lynch, 2004).

These findings emphasize the relevance of early interventions and targeted treatment as well as prevention and mental health promotion, all of which require early identification of delays or problems in child development. The assessment of behaviors, development, and cognitive functions in early childhood is thus highly significant. Especially in light of the early manifestation of externalizing disorders, researchers appeal for early interventions (Angold & Egger, 2007; Miller et al., 2002; Sonuga-Barke & Halperin, 2010).

Early identification of behavioral and emotional problems requires sound diagnostic instruments assessing potential developmental delays and symptoms of mental disorders. A requirement in assessing preschoolers’ behaviors should be to consider the high inter- and intraindividual variance of a child’s behavior and the particularities of psychopathologies in this particular age range compared with school age and adolescence. This is exemplified by the work undertaken by Abel and Hautzinger (2013), who illustrated the different characteristics of major depression in the course of development (from preschool-age to adolescence). For example, preschool children tend to demonstrate more mood swings and express their feelings or emotional problems as somatic symptoms (stomach pains or headache). Such differences should be addressed when developing a tool for assessing preschoolers’ behaviors.

However, due to rapid development between the ages of 1 and 6 years, it is challenging to assess whether a child’s behavior falls within the range of “normal” development or is a manifestation of psychopathology. That is why there is major interest in instruments focusing on young age groups that assess potential developmental delays to differentiate between normal and deviant child development. Several studies have emphasized the importance of assessing developmental status when evaluating and judging psychopathology of young children. They highlight the possibility that young children are at risk of being erroneously diagnosed with a disorder if developmental milestones are neglected. For example, Elder’s (2010) study revealed that the youngest children in a class are up to 3 times more likely to be diagnosed with attention deficit/hyperactivity disorder (ADHD) and be given psychostimulant treatment than the oldest children in the same class. Of note, teacher ratings largely depended on the children’s ages. These results were replicated in several international studies (Evans et al., 2010; Morrow et al., 2012; Schwandt & Wuppermann, 2016). On the contrary, in a Swedish study (Ulberstad & Boström, 2017) applying the Quantified Behavior Test (QbTest©; Ulberstad, 2012; which objectively assesses the three ADHD core symptoms), the differences in ADHD symptoms between the oldest and youngest children in a class disappeared when comparing the children with their respective age and gender norms. Thus, judging behavior problems in conjunction with the developmental status is crucial to diagnose young children correctly.

Although instruments for diagnosing behavioral or emotional problems in childhood have tended to be well validated and reliable (Koglin et al., 2007; Renner et al., 2004), standardized methods for assessing developmental status in early childhood are rare. Conners Early Childhood scales are one exception (Conners EC™; Conners, 2009). They are among the set of Conners questionnaires (Conners 3™ for children and adolescents aged 6–18 years; Conners, 2008a; German version: Lidzba et al., 2013; CAARS™ for patients aged 18 years and older; Conners et al., 1999; German version: Christiansen et al., 2014). The Conners EC™ scales are an internationally acknowledged assessment tool for preschool-age children (2;0–6;11 years). With different forms for parents and childcare providers, the Conners EC™ scales assess *DSM*-based current behavioral and developmental problems in children with cross-situational ratings in a multi-informant and comprehensive manner. An especially relevant advantage is the simultaneous assessment of developmental milestones, which enables concerns to be related to the child’s developmental status.

As outlined above, with such an assessment, behavioral problems can be rated and classified with respect to developmental delays, thus improving diagnostic judgments. The Conners EC™ scales are currently available in English, Spanish, and, as of recently, in German. There is ample evidence that careful review of the quality criteria, especially measurement invariance, is mandatory for transferring psychometric tests to other countries or cultures, as cross-cultural generalizability is not self-evident (Christiansen et al., 2016; Huss et al., 2001; Sperber, 2004). Thus, the present study aimed to validate the German version of the Conners EC™ (Harbarth et al., 2017) by replicating the derived factor structure of the American original for the Behavior scales and empirically confirming the theoretically assumed Developmental Milestone scales, analyzing correlational influences of sociodemographic variables (age, gender, and educational level of parents) on ratings, and assessing convergent and discriminant validity. The German adaptation of the Conners EC™ provides a broadband assessment tool for young children aged 2 to 6 years, similar to the Conners Comprehensive Behavior Rating Scales (Conners CBRS™ for children and adolescents aged 6–18 years; Conners, 2008b), with the advantage of assessing behavioral, emotional, and social concerns related to the child’s developmental status.

## Method

### Procedure and Participants

This is a cross-sectional study with German-speaking children aged 2;0 to 6;11 years who were assessed by their parents and childcare providers. The data were collected by five study centers located in the German cities of Marburg, Dortmund, Koblenz, Saarbrücken, and Rostock. As letters approached kindergartens in different counties, we cannot provide an exact response rate as not all replied; for recruitment details see Bergold et al. (2019).

After obtaining a positive ethics vote from institutional review boards, participants were recruited by convenience sampling in kindergartens and participating university clinics willing to contribute to the study, between autumn 2013 and summer 2015. Parents and childcare providers were provided with a short study description and asked to complete the German version of the Conners EC™, the German version of the Strengths and Difficulties Questionnaire (SDQ-Deu; Goodman, 2005), and a German rating scale assessing behavior in preschool children (VBV 3-6; Döpfner et al., 1993) as well as questions on child’s age, sex, and school. Written informed consent was obtained from all participants and their confidentiality was ensured. All participants completed the questionnaires at home and sent them back to the study centers. In addition, an online version of both questionnaire versions was created using the platforms UNIPARK and QuestBack, allowing the survey to be filled out personally by interested parents and childcare providers. All participating kindergartens received a 1-day workshop on ADHD as an incentive for participation.

Our total sample consisted of *N* = 795 parent and *N* = 667 childcare provider ratings. Of these, about 180 parents and 20 childcare providers filled out the questionnaire’s online version. After excluding cases revealing a pattern response and testing the assumption of missing completely at random (R. J. A. Little, 1988), we obtained a final sample of 720 parent and 599 childcare provider ratings with a balanced gender ratio of boys and girls for the parent (50% male) and childcare provider version (49% male). The majority of ratings were from mothers (84%) and female childcare providers (98%). Eighty-one children (32 girls and 59 boys) who had been clinically diagnosed with a mental disorder were recruited, although no information was available on the specific diagnosis or its frequency for data protection reasons. All diagnoses were ICD-10 based and made by trained senior staff at the clinics. We conducted no separate analyses with the clinical sample in this study; for further details on this sample, see Bergold et al. (2019). Childcare providers were not systematically informed about the children’s diagnostic status. We targeted 1-year steps for age separately for boys and girls for both forms. We managed to collect at least 40 data sets for all age and gender groups for the parent and childcare provider versions, except for 2-year-old boys and girls, and 6-year-old boys (see Table 1). The rater’s sex and parents’ educational level are presented in Table 2.

**Table 1.** Age and Sex Distribution of the German Normative Sample.

Age (years)	Parent version			Childcare provider version		
	Girls	Boys	Total	Girls	Boys	Total
2	50	60	110	32	39	71
3	85	81	166	59	55	114
4	93	93	186	84	83	167
5	92	83	175	82	81	163
6	40	43	83	45	39	84
Total	360	360	720	302	297	599

**Table 2.** Sex of Rater and Educational Level of Parents.

Rater/sex		Parent version	Childcare provider version
		84% mother 6% father 9% both 1% other caregiver	98% female 2% male
Parental educational level		Mother (%)	Father (%)
Lower educational level	Primary school	1.1	2.0
	Main school	7.8	16.0
	Middle school	30.0	22.7
Total		38.9	40.7
Higher educational level	Abitur	16.4	7.9
	University of applied sciences	18.0	22.0
	University	26.6	29.4
Total		61.0	59.3

### Measures

#### Conners EC™

The Conners EC™ scales (Conners, 2009) assess a wide range of behavioral, emotional, social, and developmental concerns in preschool children aged 2 to 6 years. It is a multi-informant assessment with versions for parents and childcare providers. In both versions, the behavior of the child is rated dimensionally in its frequency or intensity, respectively, on a 4-point Likert-type scale from 0 (*not true at all/never, seldom*) to 3 (*very much true/very often, very frequently*). The long form of the behavior scales consists of 110 items in the parent and 112 items in the childcare provider version, assessing Inattention/Hyperactivity, Defiant/Aggressive Behaviors, Social Functioning/Atypical Behaviors, Anxiety, Mood and Affect, and Physical Symptoms. In the original version, the items on the Mood and Affect, Physical Symptoms, and Atypical Behavior scales did not form independent factors in the exploratory factor analysis (EFA) of the pilot data phase and were regarded as “rational scales,” which were “retained due to their theoretical and clinical significance” (Conners, 2009).

The Developmental Milestone scales comprise 75 items (70 items in the version for childcare providers) that are rated on a 3-point Likert-type scale from 0 (*no/never or rarely*) to 2 (*yes/always or almost always*). The scales assess the child’s developmental status in five key areas of early child development, namely, Adaptive Skills, Communication, Motor Skills, Play, and Pre-academic/Cognitive Skills. The Conners EC™ also features Other Clinical Indicators as screener items for PTSD (post-traumatic stress disorder), Specific Phobia, Tics, Trichotillomania, Pica, Self-Injury, Stealing, Cruelty to Animals, Fire Setting, and Perfectionism. If answered positively (>85th percentile of the normative sample), they indicate the need for further clarification.

In addition, the Conners EC™ measures overly positive, negative, or inconsistent response styles, through three validity scales, and helps estimate whether the questionnaires were filled out validly by their raters. All Conners EC™ scales were translated into German according to the translation guidelines from Multi-Health Systems (MHS Inc.) and then back translated. Norms for a German-speaking sample were also established (Harbarth et al., 2017).

#### Questionnaire for assessing preschool children’s behavior

The *Verhaltensbeurteilungsbogen für Vorschulkinder* (VBV 3-6; Döpfner et al., 1993) is a German questionnaire for assessing behavioral problems and skills of children from 3 to 6 years of age. Parents or childcare providers are instructed to rate the frequency of a child’s behavior over the past 4 weeks from 0 (*never*) to 4 (*very often*). The VBV 3-6 in the parent version consists of 53 items and the childcare provider version, 93 items. Both versions assess social-emotional skills, defiant-aggressive behaviors, inattention and hyperactivity versus perseverance in play, as well as emotional concerns. The German normative sample consists of 392 preschool children. Psychometric properties are generally considered as good and exhibit good to satisfactory internal consistency (Renner et al., 2004). The internal consistency in this study was good to excellent (.86 ≤ α ≤ .94) in the childcare provider and parent versions with the exception of social-emotional skills (α = .65) and emotional concerns (α = .78).

#### SDQ–German version

The German version of the SDQ (SDQ-deu; Goodman, 2005) is a standard, widely used screening instrument for children aged 4 to 16 years. A total of 25 items assess emotional problems, behavior problems, hyperactivity, behavior problems with peers, and prosocial behavior. The child’s behavior over the past 6 months is rated on a 3-point Likert-type scale ranging from 0 (*not true*) to 2 (*certainly true*) by parents or childcare providers. A total score is obtained by summing up the item scores, excluding the prosocial behavior items. The German version of the SDQ is well validated and has a normative sample of 930 children. Internal consistencies in the original instrument range from α = .58 to α = .82. In our study, internal consistency ranged from α = .61 (behavior problems, parent version) to α = .86 (hyperactivity, childcare provider version).

#### Observation sheet for preschool children

The *Beobachtungsbogen für Kinder im Vorschulalter* (BBK 3-6; Frey et al., 2008) is an observation sheet used as a screening instrument for the global development of a child aged 3 to 6 years and assesses 12 different skills and functioning areas: task orientation, initial reading, calculating, writing, communication, reflexivity, language development, understanding of the literature, fine motor skills, gross motor skills, media competence, intensity of play, aggression, and shyness. Based on a normative sample of 3,456 children, a development profile can be compiled and reference values used to obtain initial indications as to whether a child has a developmental risk or special talent. Our study’s internal consistency ranged from α = .75 (gross motor skills) to α = .95 (language development), which reflects findings from the original (Frey et al., 2008).

### Statistical Analyses

All raw data were stored in a database in Marburg, Germany (Department of Clinical Child and Adolescent Psychology at the Philipps University Marburg,). Data reduction and analyses were carried out using the Statistical Package or Social Science Version 20 (IBM Corp, 2011) and M*plus* Version 7 (Muthén & Muthén, 2015).

First, to not blindly assume the American original factor structure, but to identify the optimal factor structure of the German normative sample, a series of EFAs was conducted. Regardless of the American model, this procedure allows a valid exploration of the factor structure and item loadings to identify the optimal model for the German normative sample. According to Conners (2008) and Christiansen et al. (2016), items were included if they loaded significantly (>.35) on a given factor and cross-loaded lower than .35 on all other factors. Within the principal component analysis, the orthogonal rotation method was used for the behavior scales to identify uncorrelated factors. In contrast, we used oblique rotation methods for the developmental milestone scales to allow correlations between factors due to our theoretical assumptions. The scree test and eigenvalues (>1.0) were used to identify the number of factors for rotation. The final method used to identify the most suitable factor solution for the German sample was Horn’s (1965) parallel analysis. In the case of high correlations between the extracted factors, a second-order factor analysis was conducted to analyze whether there are first-order factors that could be merged into second-order ones. With Cronbach’s alpha (Cronbach, 1951), we investigated the internal consistency of the postulated factors in the different questionnaire versions, with .7 ≤ α < .9 indicating acceptable and α ≥ .90 indicating excellent internal consistency (Bland & Altman, 1997).

Second, the American model of the empirical behavior scales (Inattention/Hyperactivity, Social Functioning/Atypical Behaviors, Defiant/Aggressive Behaviors, and Anxiety) was also tested with the data of the German normative sample (same data as in the EFA). For this purpose, items of the Conners EC™ were grouped into “parcels” based on intercorrelations analogously to the procedure of the standardization of the original version (see Cattell & Burdsal, 1975; Conners, 2009; Hughey & Burdsal, 1982; T. D. Little et al., 2013) and then used for the confirmatory analyses. Maximum likelihood estimation (MLR) was used for estimation (Brown, 2006). To date, there are no empirically verified model assumptions about the original version’s Developmental Milestone scale. Using the balancing approach (see T. D. Little et al., 2013), items were initially grouped into parcels and then used for confirmatory factor analyses (CFAs) restricted to the five postulated and theory-based factors of the original version, with correlations allowed between each of them. We additionally tested a hierarchical model (five factors subsumed under a global factor “general development”) to examine and compare the model fits. Comparisons between the two models were made by computing the χ^2^ difference test (α = 95%). According to the recommended procedure in the literature, we calculated several model fit indices to evaluate the results of our analyses (Bühner, 2006; Hu & Bentler, 1999; Schermelleh-Engel et al., 2003): comparative fit index (CFI), Tucker–Lewis index (TLI), root mean square error of approximation (RMSEA), and standardized root mean square residual (SRMR). Marsh et al. (2004) proposed that “conventional CFA goodness of fit criteria are too restrictive when applied to most multifactor rating instruments” (p. 325). With respect to this, CFI values ≥.95 represent a good model fit relative to the independence model. The TLI, also known as nonnormed fit index (NNFI; Bearden et al., 1982; Tucker & Lewis, 1973), measures relative fit und indicates an acceptable model fit with values larger than .95, although values larger than .90 are interpreted as acceptable fit. An RMSEA between .08 and .10 can be considered as a “mediocre” fit, whereas values ≤.05 indicate a good fit (Schermelleh-Engel et al., 2003). The SRMR is a measure of the average of the standardized residuals between the observed and hypothesized covariance matrices (Chen, 2007). Values ≤.10 indicate an acceptable model fit and values ≤.05 indicate a good model fit (Hu & Bentler, 1999). Again, Cronbach’s alpha was calculated for the scales used for CFA.

Third, differences in subscale ratings were calculated for age, gender, and parents’ educational degree, with multivariate analyses of variance (MANOVAs) to analyze associations between those sociodemographic variables and symptom ratings. To this end, five groups were formed according to age: 2 years, 3 years, 4 years, 5 years, and 6 years. For educational level, parents were asked to rate their highest level of education attained according to the German education system, “university (of applied science) degree” = highest educational level followed by Abitur = highest school leaving qualification in Germany and “lower educational level.” All Behavior scales (separated into empirical and theory-based ones), Developmental Milestone scales, and validity scales of the parent and childcare provider version were reviewed. In the case of significant results of multivariate analyses, post hoc analyses for pairwise comparisons were conducted.

To test the convergent and discriminant validity of the Conners EC™, the Behavior scales as well as the Developmental Milestone scales were correlated with scales of the SDQ-deu (Goodman, 2005), the VBV 3-6 (Döpfner et al., 1993), and the BBK 3-6 (Frey et al., 2008). A series of Pearson correlations was performed to assess convergent and discriminant validity.

## Results

### EFA

Detailed information on the simple structure (EFA) of the Conners EC™ is available as Supplementary Material accompanying the online article (Tables S3–S6).

#### Parent version

Results of the EFA of the parent version revealed eight factors accounting for 41.20% of the total variance: Inattention/Hyperactivity, Defiance/Temper, Atypical Behaviors, Aggressive Behaviors, Social Functioning, Anxiety, Sleep Problems, and Physical Symptoms (.70 ≤ α ≤ .94). In contrast to the American model (four empirically extracted factors), we identified two other factors, namely, Sleep Problems and Physical Symptoms. In contrast to Conners’ (2009) findings, who identified the two scales “Social Functioning/Atypical Behaviors” and “Defiant/Aggressive Behaviors,” our EFA resulted in the extraction of four separated factors: “Social Functioning,” “Atypical Behaviors,” “Defiance/Temper,” and “Aggressive Behaviors.” The EFA of the Developmental Milestone scales of the parent version resulted in a four-factor solution explaining 54.13% of total variance: Adaptive Skills, Communication, Motor Skills, and Play (.65 ≤ α ≤ .94). We detected moderate to high correlations between the factors. Those four factors were subjected to a second-order factor analysis with the orthogonal rotation method as we assumed no conceptual coherence between second-order factors. One global second-order factor was identified, explaining 54.02% of total variance (Cronbach’s α = .96). This scale includes all developmental milestone items and provides a comprehensive global assessment of the development of a child in relation to his or her age and sex.

#### Childcare provider version

Our analyses of the childcare provider version resulted in a four-factor solution explaining 40.70% of the total variance. These factors corresponded to the empirical factors in the American version: Inattention/Hyperactivity, Defiant/Aggressive Behaviors, Social Functioning/Atypical Behaviors, and Anxiety (.87 ≤ α ≤ .92). Analogically to the parent version, the EFA of the Developmental Milestone scales revealed a four-factor solution explaining 61.85% of total variance (.58 ≤ α ≤ .96) and a global second-order factor explaining 49.35% of total variance containing all items of the Developmental Milestone scales (Cronbach’s α = .97). See Table 3 for detailed results on the EFA analyses.

**Table 3.** Results of Exploratory Factor Analyses of Conners EC™ Parent Version and Childcare Provider Version.

Behavior scales				Developmental milestone scales			
Factor	%	No.	α	Factor	%	No.	α
Conners EC™ parent version							
1	21.71 (15.41)	14 (0.35–0.95)	.940	1	39.45 (15.78)	15 (0.36–0.96)	.942
2	4.51 (3.20)	12 (0.31–0.87)	.883	2	6.98 (2.79)	12 (0.50–0.84)	.928
3	3.98 (2.83)	10 (0.32–0.68)	.769	3	4.56 (1.82)	4 (0.68–0.85)	.835
4	3.54 (2.52)	6 (0.37–0.81)	.780	4	3.46 (1.39)	6 (0.36–0.83)	.647
5	2.04 (1.45)	8 (–0.67–[–0.37])	.759	Second-order factor	54.02 (2.70)	40 (0.52–0.86)	.957
6	2.20 (1.56)	14 (0.31–0.50)	.781				
7	1.72 (1.22)	4 (0.31–0.85)	.703				
8	1.50 (1.07)	3 (0.51–0.76)	.721				
Conners EC™ childcare provider version							
1	25.25 (18.18)	16 (–0.41–0.91)	.924	1	48.24 (21.71)	18 (0.37–1.00)	.962
2	6.08 (4.38)	21 (0.31–0.79)	.916	2	6.24 (2.81)	11 (0.46–1.09)	.950
3	5.14 (3.70)	18 (–0.80–0.60)	.869	3	3.97 (1.79)	9 (0.43–0.99)	.905
4	4.25 (3.06)	17 (0.32–0.79)	.874	4	3.41 (1.54)	3 (0.55–0.74)	.575
				Second-order factor	49.35 (2.96)	45	.974

*Note*. EC = early childhood; % = total variance and factor variance explained; No. = number of items and range of eigenvalues in parentheses; α = Cronbach’s alpha.

For further evidence of the assumed multidimensionality of the factor structure, all scales in both versions of the Conners EC™ were intercorrelated (for details see Tables S1 and S2 in the Supplementary Material). These moderate correlations reveal the multidimensional factor structure. The intercorrelation of Defiant/Aggressive Behaviors and Social Functioning/Atypical Behaviors scales with their subscales is very high, as expected. Furthermore, all Developmental Milestone scales revealed high correlations among each another, as we had assumed.

### CFAs

With regard to our intention to adapt the American model, all parcels including all original items were used to test the factor model of the empirical behavior scales as well as the Developmental Milestone scales of the parent and childcare provider version with the German sample. Table 4 details the model fits of the CFAs for the empirical behavior scales and the Developmental Milestone scales with the first-order and the hierarchical models.

**Table 4.** Model Fit Parameters of Confirmatory Factor Analyses.

GFI	Behavior scales		DM scales (first-order model)		DM scales (hierarchical model)	
	Parent	Childcare provider	Parent	Childcare provider	Parent	Childcare provider
*df*	71	59	117	105	116	105
χ^2^ test	497.70 *p* < .001	465.25 *p* < .001	460.94 *p* < .001	375.77 *p* < .001	457.02 *p* < .001	375.77 *p* < .001
CFI	.93	.93	.97	.97	.97	.97
TLI	.91	.90	.96	.96	.96	.96
RMSEA	.09	.11	.06	.06	.06	.06
SRMR	.05	.05	.03	.02	.03	.02

*Note*. GFI = goodnes of fit index; DM = Developmental Milestone scales; *df* = degrees of freedom; CFI = comparative fit index; TLI = Tucker–Lewis index; RMSEA = root mean square error of approximation; SRMR = standardized root mean square residual.

#### Parent version

Our CFA results indicated an acceptable to good fit of the postulated factor structure of the Behavior scales in the parent version. All model parameters of the Developmental Milestone scales (parent and childcare provider version) suggested an acceptable to good model fit. By analyzing the two proposed models (first order vs. hierarchical order) with the χ^2^ difference test in the parent version, we note that the hierarchical model has a significantly better fit (Δχ^2^ = 460.94–457.02, *df* = 117–116, χ^2^_calc_ = 3.92 > χ^2^_crit_ = 3.84) on the German data than the first-order model without the second-order factor “general development.” Cronbach’s alpha of the German version showed values largely comparable to those of the American original version. Cronbach’s alpha was acceptable for all behavior scales in the parent version (α ≥ .70), with the exception of Physical Symptoms (.68) and Sleep Problems (.57). Cronbach’s alpha for the individual Developmental Milestone scales was excellent (α ≥ .90), with the exception of Play (.83). The Global Development scale (including all Developmental Milestone items) demonstrated excellent internal consistency of .97.

#### Childcare provider version

A similar scenario appeared in the CFA of the childcare provider version of the Conners EC™. All model parameters in the childcare provider version’s behavior scales resulted in satisfactory to good model fit values with the exception of RMSEA (see Table 4). The CFA results of the Developmental Milestone scales (both models) indicated good model fits. No significant difference was identified between the two models with χ^2^ difference test in the childcare provider version, whose analysis of the behavior scales’ internal consistency resulted in values in an acceptable range (70 ≥ α ≥ .95), with the exception of Physical Symptoms (.68). Cronbach’s alpha for the individual Developmental Milestone scales was good to excellent (.80 ≤ α ≤ .98), as was Global Development’s (.98).

With regard to the Physical Symptoms scale, item difficulty analyses showed an increased Cronbach’s alpha (parent version .70 and childcare provider version .72) when excluding questions about eating behavior (“Eats too much” and “Eats too little”). However, these two items were retained to preserve international comparability. See Table S7 for details on internal consistency in the Supplementary Material.

### Normative Data

A series of sex by age group analyses was conducted with each of the Conners EC™ scales as the dependent variable for both versions. For a detailed overview of univariate effects of age and sex on Conners EC behavior scales and Developmental Milestone scales, see Tables S8 and S9 (parent version) and Tables S10 and S11 (childcare provider version) in the Supplementary Material.

#### Parent ratings

In the parent version, the empirical scale Defiant/Aggressive Behaviors, *F*(4, 692) = 3.63, *p* = .006, $\eta_{p}^{2}$ = .021; as well as the subscales Defiance/Temper, *F*(4, 692) = 3.68, *p* = .006, ηp^2^ = .021; and Aggressive Behaviors, *F*(4, 692) = 4.60, *p* < .001, $\eta_{p}^{2}$ = .026, exhibited significant associations with age (see Table 5). Older children were rated as displaying fewer behavioral problems than younger ones. Analyses of the Developmental Milestone scales yielded a significant main effect of age, of medium-to-large size (.068 ≤ $\eta_{p}^{2}$ ≤ .315), on the subscales as well as on the Global Development scale, *F*(4, 692) = 65.68, *p* < .001, $\eta_{p}^{2}$ = .275, with older children rated lower (i.e., older children had reached more milestones).

**Table 5.** MANOVA of Sex and Age on Conners EC™ Parent Version and Childcare Provider Version.

Conners EC™ scales	Source	Wilks’s lambda	*F*(*df*)	*p*	$\eta_{p}^{2}$
Conners EC™ parent version					
Empirical scales	Sex	.96	8.05 (4, 692)	<.001	.044
	Age	.94	2.50 (16, 2115)	<.001	.014
	Sex × Age	.98	0.71 (16, 2115)	.787	.004
Subscales	Sex	.91	14.23 (5, 690)	<.001	.093
	Age	.93	2.53 (20, 2289)	<.001	.018
	Sex × Age	.97	0.98 (20, 2289)	.490	.007
Theoretical assumed scales	Sex	.99	2.64 (3, 702)	.049	.011
	Age	.98	1.43 (12, 1858)	.145	.008
	Sex × Age	.97	1.68 (12, 1858)	.066	.009
Developmental Milestone scales	Sex	.97	3.51 (6, 696)	.002	.029
	Age	.57	17.53 (24, 2429)	<.001	.130
	Sex × Age	.98	0.69 (24, 2429)	.863	.006
Conners EC™ childcare provider version					
Empirical scales	Sex	.94	9.26 (4, 560)	<.001	.062
	Age	.93	2.53 (16, 1711)	.001	.018
	Sex × Age	.99	0.49 (16, 1711)	.954	.003
Subscales	Sex	.95	8.20 (4, 567)	<.001	.055
	Age	.96	1.43 (16, 1733)	.117	.010
	Sex × Age	.98	0.85 (16, 1733)	.634	.006
Theoretical assumed scales	Sex	.99	3.08 (2, 582)	.047	.010
	Age	.99	1.13 (8, 1164)	.337	.008
	Sex × Age	.97	2.10 (8, 1164)	.033	.014
Developmental Milestone scales	Sex	.96	3.82 (6, 538)	.001	.041
	Age	.26	37.32 (24, 1878)	<.001	.288
	Sex × Age	.95	1.06 (24, 1878)	.387	.012

*Note. df* = degrees of freedom; EC = early childhood.

Sex was associated significantly with these scales: Inattention/Hyperactivity, *F*(1, 715) = 9.52, *p* = .002, $\eta_{p}^{2}$ = .013; Defiant/Aggressive Behaviors, *F*(1, 715) = 16.30, *p* < .001, $\eta_{p}^{2}$ = .022; Social Functioning/Atypical Behaviors, *F*(1, 715) = 15.41, *p* < .001, $\eta_{p}^{2}$ = .021; and Physical Symptoms, *F*(1, 715) = 3.85, *p* < .05, $\eta_{p}^{2}$ = .025; and the subscales, Defiance/Temper, *F*(1, 715) = 4.66, *p* = .031, $\eta_{p}^{2}$ = .006; Aggressive Behaviors, *F*(1, 715) = 49.97, *p* < .001, $\eta_{p}^{2}$ = .065; Social Functioning, *F*(1, 715) = 16.30, *p* < .001, $\eta_{p}^{2}$ = .022; Atypical Behaviors, *F*(1, 715) = 5.64, *p* = .018, $\eta_{p}^{2}$ = .008; and Negative Impression, *F*(1, 715) = 13.42, *p* < .001, $\eta_{p}^{2}$ = .018. Generally, girls received lower ratings, except for the Physical Symptoms subscale. All individual Developmental Milestone scales and the Global Development scale ( $\eta_{p}^{2}$ = .015) were also significantly associated with sex with small effects (.014 ≤ $\eta_{p}^{2}$ ≤ .021). Girls tended to attain developmental milestones earlier than boys.

All scales correlated significantly with the mother’s level of educational achievement as reflected by small effects in the MANOVA (.024 ≤ $\eta_{p}^{2}$ ≤ .031), with children from mothers with higher educational levels receiving lower scores. This corresponds to the results of the paternal level of education as the MANOVA resulted in effects on the behavior and Developmental Milestone scales in the parent version and on all behavior scales in the childcare provider version with consistently small effect sizes (.025 ≤ $\eta_{p}^{2}$ ≤ .026).

#### Childcare provider ratings

In the childcare provider version, younger children (2–3 years old) tended to be rated higher (more concerns) on the Inattention/Hyperactivity scale relative to older children, *F*(4, 535) = 3.66, *p* < .006, $\eta_{p}^{2}$ = .027. All Developmental Milestone scales were also significantly associated with age (.232 ≤ $\eta_{p}^{2}$ ≤ .607), as was the Global Development scale, *F*(4, 535) = 185.67, *p* < .001, $\eta_{p}^{2}$ = .581, with the older children being rated as having reached more milestones.

Inattention/hyperactivity, *F*(1, 526) = 18.01, *p* < .001, $\eta_{p}^{2}$ = .033; Defiant/Aggressive Behaviors, *F*(1, 526) = 9.08, *p* = .003, $\eta_{p}^{2}$ = .017; Social Functioning/Atypical Behaviors, *F*(1, 526) = 20.02, *p* < .001, $\eta_{p}^{2}$ = .037, as well as the subscales Aggressive Behaviors, *F*(1, 526) = 16.62, *p* < .001, $\eta_{p}^{2}$ = .031; Social Functioning, *F*(1, 526) = 19.60, *p* < .001, $\eta_{p}^{2}$ = .036; and Atypical Behaviors, *F*(1, 526) = 10.21, *p* < .001, $\eta_{p}^{2}$ = .019, were significantly influenced by sex, with boys being given higher pathology ratings. All Developmental scales and the Global Development scale yielded a significant main effect of small size (.009 ≤ $\eta_{p}^{2}$ ≤ .021), with girls being rated as having reached more Developmental Milestones than boys. Although the MANOVA resulted in a significant Age × Sex interaction, *F*(8, 1164) = 2.10, *p* = .033, $\eta_{p}^{2}$ = .014, no significant association was apparent at the univariate level.

Corresponding to the parent version, all scales were associated significantly with the mother’s level of educational achievement as reflected by small effects in the MANOVA (.025 ≤ $\eta_{p}^{2}$ ≤ .032). Only the Behavior scales in the childcare provider version were associated with the paternal level of education as the MANOVA resulted in significant effects with small effect sizes (.033 ≤ $\eta_{p}^{2}$ ≤ .036).

### Convergent and Discriminant Validity

Table 6 illustrates our convergent and discriminant validity results. Overall, Pearson correlations revealed meaningful patterns of convergence and divergence. Scales in the Conners EC™ and those in the SDQ, VBV, and BBK designed to assess similar constructs correlated positively. Associations were moderate to high. By contrast, scales designed to measure different constructs correlated to a lesser degree. We used the subscale Prosocial Behavior for the assessment of discriminant validity.

**Table 6.** Correlations of the Conners EC™ Scales With Related VBV, SDQ, and BBK Scales.

Conners EC™ scales	Dimensions of SDQ^a^, VBV^b^, or BBK^c^	Parent^d^	Childcare provider
Behavior scales			
Inattention/Hyperactivity	SDQ Hyperactivity	.85**	.88**
	VBV Attention Deficit/Motor Agitation	.85**	.83**
	VBV Hyperactivity versus Perseverance in Play	.83**	.90**
	*SDQ Prosocial Behavior*	*–.37* **	*–.48* **
Defiant/Aggressive Behaviors	SDQ Behavior Problems	.70**	.72**
	VBV Impulsive and Defiant Behaviors Against Parents/Childcare Provider	.81**	.74**
	VBV Defiant-Aggressive Behaviors	.84**	.83**
	VBV Verbal Physical Aggression Against Siblings/Verbal Aggression against Other Children	.72**	.74**
	*SDQ Prosocial Behavior*	*–.40* **	*–.48* **
Social Functioning/Atypical Behaviors	SDQ Behavior Problems with Peers	.60**	.65**
	VBV Interplay and Communication Skills	—^e^	−.67**
	*SDQ Prosocial Behavior*	*–.50* **	*–.61* **
Anxiety	SDQ Emotional Problems	.68**	.62**
	VBV Emotional Lability	.60**	.51**
	VBV Emotional Concerns	.60**	.57**
	*SDQ Prosocial Behavior*	*–.18* **	*–.13* *
Mood and Affect	SDQ Emotional Problems	.51**	.53**
	VBV Emotional Lability	.57**	.58**
	*SDQ Prosocial Behavior*	*–.30* **	*–.40* **
Developmental Milestone scales^f^			
Communication	BBK Language Development	−.54**	−.54**
Motor Skills	BBK Fine Motor Skills	−.52**	−.59**
	BBK Gross Motor Skills	−.32*	−.52**
Play	BBK Intensity of Play	−.36*	−.10
Pre-academic	BBK Initial Reading, Calculation, and Writing	−.80**	−.78**

*Note*. Correlations of discriminant validity are shown in italic. EC = early childhood; VBV = Questionnaire for Assessing Preschool Children’s Behavior; SDQ = Strengths and Difficulties Questionnaire–German version; BBK = observation sheet for preschool children; DM = Developmental Milestone scales.

a *N* = 409–512. ^b^*N* = 408–515. ^c^*N* = 40–43. ^d^*r* = Pearson’s rank correlation coefficient. ^e^Parent rating (VBV) do not assess “Interplay and Communication Skills.” ^f^Items of DM scales were inverted for statistical analyses.

* *p* < .05. **p < .01.

## Discussion

In the present study, we analyzed the factor structure of the German adaptation of the Conners EC™ scales (Conners, 2009) in a large sample of parents and childcare providers of German-speaking children through exploratory and confirmatory analyses, identified correlational influences of sociodemographic variables on parent and childcare provider ratings, and determined construct validity by correlating the Conners EC™ scales with several other well-validated German measures assessing preschoolers’ behaviors.

Overall, we confirmed the factor structure of the American original of the Conners EC™ within the German validation. The conformity of the Conners EC™ scales with scales of other commonly used instruments in the German-speaking population provides evidence for an adequate assessment of the assumed constructs of behaviors in preschool-age children. The German normative sample consists of 720 parental data and 599 childcare provider data and reveals a sufficiently high number of assessments in the age and gender distribution—a significant strength of this study. Our study contributes to the assessment of behaviors in preschool-age children as it provides a large data set to compare behaviors and developmental levels assessed by parents and childcare providers. At this point, allow us to emphasize our large data set of childcare providers. To our knowledge, no studies to date have investigated such a comprehensive assessment tool, focusing on such a wide range of behavioral, emotional, and social concerns of German preschool children (e.g., SDQ; Rogge et al., 2018).

Exploratory analyses of the parent and childcare provider ratings identified the four behavior scales (Inattention/Hyperactivity, Defiant/Aggressive Behaviors, Social Functioning/Atypical Behaviors, and Anxiety) within the German sample. Unlike the American original, we provide psychometric evidence for theoretically assumed factors in the behavior scales as we identified two further scales in the parent version, namely, Sleep Problems and Physical Symptoms. We thus maintain that a differentiated assessment of somatic symptoms in early childhood with the Conners EC™ is appropriate, not least because physical symptoms are given high priority in childhood depression and anxiety disorders (Whalen et al., 2017). Furthermore, the Defiance/Temper and Aggressive Behaviors, as well as Social Functioning and Atypical Behaviors scales resulted in independent scales in the parent version. In the childcare provider version, the EFA provided no independent factor of Physical Symptoms, and Sleep Problems are not assessed in that version. Instead, Anxiety, Mood and Affect, as well as items assessing Pain loaded on one factor in the childcare provider version that can be characterized as “internalizing problems.” A possible explanation for these results may be the childcare providers’ inadequate discrimination of internal problems, which are less obvious than externalizing behaviors (e.g., defiance or hyperactivity). These findings support previous research on the comparison of parent and teacher reports of internalizing symptoms (see Bergold et al., 2019; Grietens et al., 2004).

Our CFAs with the empirical behavior scales resulted in an at least acceptable model fit, replicating the factor structure of the American original scales. Cronbach’s alpha for all scales of the parent and childcare provider version was acceptable to high, with the exception of Physical Symptoms and Sleep Problems (parent version only). These findings support the suggestion for a more differentiated assessment of physical symptoms in preschool age (eating, sleep, and pain) rather than aggregating the items within one “global” scale.

Regarding the Developmental Milestone scales, the German normative sample’s EFA results differ from the American original in the parent and childcare provider form. We extracted four scales with a hierarchical second-order factor that we termed “general development.” A closer inspection of the four extracted factors (Adaptive Skills, Communication, Motor Skills, and Play) in the parent version showed that we were unable to extract the “fifth” scale Pre-academic/Cognitive Skills of the theoretically assumed American original factor structure because the corresponding items loaded significantly on the other four factors. This finding has important implications for understanding and defining developmental “milestones,” as pre-academic and cognitive skills can be understood as more global, which require specification in motor or communication skills, for example. A closer look at the items in the Pre-academic/Cognitive Skills scale revealed that most of them are content-related and very similar to the other assessed areas (e.g., Item 33 “Compares objects using the concepts heavier/lighter and bigger/smaller” or Item 53 “Names most body parts [including shoulder, elbow, wrist, and knee], which loaded strongly on the ‘communication’ scale.”) Due to our defined exclusion criteria of items, which state that no high cross-loadings were permitted onto more than one factor, many of the items in the original Pre-academic/Cognitive Skills scale had to be excluded for EFA. This indicates a conceptual problem as the excluded items might fit conceptually to one of the other Developmental Milestone scales. In the childcare provider version, further inspection of the extracted scales revealed a similar pattern; note that we failed to identify the theoretically assumed scales by Conners (see Table S11).

With regard to measurement invariance, we provide initial evidence for the theoretically assumed Behavior scales and Developmental Milestones scales as the American original has not been subjected to psychometric validation (see Conners, 2009). We did not aim to empirically compare the German with the American normative sample findings. We tested this theoretically latent model for the first time by conducting exploratory and confirmatory analyses with all Conners EC™ scales. For this purpose, configural, metric, and scalar invariance were not testable as we had no data from the American normative sample testing all scales. Despite the differences in the German normative sample in our EFAs from the American original’s theoretically assumed factor structure, our CFAs’ results demonstrated an overall satisfactory to good model fit. Cronbach’s alpha values were good to excellent. We can therefore assume that parent and childcare provider ratings according to those structures are well justified. Taken together, our findings reveal the adaptability of the original Conners EC™ scales. The χ^2^ difference test yielded a significantly better fit of the hierarchical model only for the parent version, but we decided to include the second-order factor “global development” to the German version due to our exploratory findings to thus enable the two rater versions to be compared.

Sex and age of the rated children as well as their parents’ educational levels were associated with the Behavior and Developmental Milestone scales. Girls were rated as exhibiting less problematic behavior across all Conners EC™ subscales and as having reached more Developmental Milestones than boys (according to parents and childcare providers). This replicates the findings of the American original version (Conners, 2009) and is in line with the particular risk factors for psychopathology in childhood described in the literature (Costello et al., 2011; Klasen et al., 2016). Those studies suggest that girls might be more compliant and behave more appropriately already in preschool years, resulting in more favorable behavior ratings. It may be that our findings provide an indication of differences in the development of mental disorders in young childhood with regard to the child’s sex as boys exhibit more early onset externalizing disorders, with girls tending to develop more internalizing disorders (Ihle & Esser, 2002; Klasen et al., 2016).

Turning to the correlational influence of age, parents and childcare providers rated older children as exhibiting fewer problematic behaviors and more advanced development than younger children. The observed association between age and the Developmental Milestone scales contributes to the validity of our adapted scales, as we show that the developmental status changes significantly depending on age. In the childcare provider version, younger children tended to be rated higher on the Inattention/Hyperactivity scale (small effect), whereas the parent version revealed no association with age. Exactly the opposite was the case with the Defiant/Aggressive Behaviors scale. Although our correlational analyses of age with that aforementioned scale yielded only small effect sizes, it underlines the importance of a multi-informant assessment of child behaviors in different living environments to make valid clinical decisions possible. Several working groups have provided different explanations for differences between raters, such as different individual standards and disparate frames of reference due to the environment and genuine variability in child’s behavior (home vs. school setting; Achenbach et al., 1987; De Los Reyes et al., 2015; Grietens et al., 2004). However, these results enhance our scales’ validity and demonstrate the most important aspect of assessing a child’s behavior in relation to their developmental status.

Taken together, the observed associations between sex and age in our normative sample contribute to the validity of the German-adapted scales because we considered sample-dependent differences.

### Limitations and Future Directions

Several key limitations must be mentioned. When doing psychometrics on a scale, the sample of participants chosen to complete the scale should be generally similar to the population the scale was originally designed for (parents and childcare providers of preschool-age children). Although we assessed a large German-speaking sample from all over Germany, our sample’s representativeness is not a given. There is a significant proportion of parents with above-average educational levels within our sample, which may be because our online sample was primarily recruited through mailing lists from three large German universities (Marburg, Dortmund, and Koblenz-Landau) which would have contributed to a significantly higher number of participants with a higher education level. In addition, there was no survey of specific sociodemographics such as ethnic background or the participating families’ native language to make assumptions about linguistic comprehensibility for nonnative speakers. Therefore, future research should capture these data to make assertions about possible effects.

Second, we established a different factor structure of the Developmental Milestone scales in our exploratory analyses. Although we extracted four factors in the parent version (Adaptive Skills, Communication, Motor Skills, and Play), the childcare provider version’s results revealed a completely different factor structure than the theoretically assumed, original American scales. Nonetheless, CFAs resulted in satisfactory to good model fits arguing for the use of the American scale in the German adaptation for the sake of international comparability.

To provide more in-depth analyses of convergent and discriminant validity, future studies could apply multitrait-multimethod analyses (MTMM; D. T. Campbell & Fiske, 1959).

## Conclusion

This is the first study empirically investigating the theoretically assumed behavior scales and Developmental Milestone scales in the American original Conners EC™. The purpose of our study was to validate the German version of the Conners EC™ through explorative and confirmatory analyses of the original American scales, analyzing associations between sociodemographic variables and symptom ratings, and to test construct validity. Our results demonstrate that the German version of the Conners EC™ possesses good overall factorial validity. Our results of the behavior scales are in line with the findings for the American original of the Conners EC™. Although our EFAs of the Developmental Milestone scales yielded factors different from the theoretically assumed original scales, CFAs showed acceptable to good model fits, so that, with respect to international studies, use of the American factor structure is justified and can be recommended to facilitate international research on psychopathology in early childhood.

## Supplemental Material

Supplemental_Material_ – Supplemental material for Do German Children Differ? A Validation of Conners Early Childhood™ Click here for additional data file. Supplemental material, Supplemental_Material_ for Do German Children Differ? A Validation of Conners Early Childhood™ by Selina Türk, Simon Harbarth, Sebastian Bergold, Ricarda Steinmayr, Eva Neidhardt, Inge Kamp-Becker, Monika Equit, Katja Wunsch and Hanna Christiansen in Journal of Attention Disorders
