# Supplementary material for: Do German Children Differ? A Validation of Conners Early Childhood™
Source: J Atten Disord. 2020 Mar 14;25(10):1441–54. doi: 10.1177/1087054720907955 (PMC8273533; doi:10.1177/1087054720907955)
Supplement: Supplemental_Material_ – Supplemental material for Do German Children Differ? A Validation of Conners Early Childhood™ [file Supplemental_Material_.pdf]

Table S1

Intercorrelations of the Conners EC^{TM} parent version scales

Scales	1	2	3	4	5	6	7	8	9	10	11	12	13	14	15	16
Behavior scales																
Inattention/ Hyperactivity																
Defiant/Aggressive Behaviors	.64**															
Defiance/Temper	.61**	.96**														
Aggressive Behaviors	.52**	.79**	.58**													
Social Functioning/ Atypical Behaviors	.55**	.47**	.41**	.45**												
Social Functioning	.40**	.33**	.28**	.34**	.89**											
Atypical Behaviors	.55**	.48**	.44**	.43**	.79**	.44**										
Anxiety	.48**	.49**	.50**	.31**	.42**	.25**	.49**									
Mood and Affect	.63**	.81**	.84**	.51**	.54**	.37**	.57**	.65**								
Physical Symptoms	.41**	.40**	.40**	.28**	.35**	.20**	.42**	.71**	.51**							
Sleep Problems	.44**	.37**	.38**	.24**	.33**	.20**	.37**	.71**	.45**	.83**						
Developmental Milestone scales																
Adaptive Skills	.16**	.10**	.12**	.02	.23**	.25**	.15**	.09*	.10**	.08*	.14**					
Communication	.27**	.08*	.09*	.03	.37**	.39**	.22**	.09*	.12**	.12**	.17**	.67**				
Motor Skills	.18**	.11**	.15**	.00	.21**	.22**	.14**	.07	.11**	.08*	.13**	.85**	.69**			
Play	.25**	.15**	.15**	.10**	.35**	.38**	.19**	.09*	.12**	.11**	.15**	.57**	.68**	.59**		
Pre-Academic/ Cognitive Skills	.21**	.10**	.13**	.01	.22**	.25**	.13**	.06	.11**	.09*	.15**	.78**	.80**	.86**	.63**	
Global Development	.23**	.11**	.14**	.02	.29**	.31**	.18**	.08*	.12**	.10**	.16**	.90**	.86**	.93**	.72**	.95**

Note. Pair-wise deletion of missing cases was used (N = 790-795).

** = p < .01; * = p < .05.

Table S2

Intercorrelations of the Conners EC™ childcare-provider version scales

Scales	1	2	3	4	5	6	7	8	9	10	11	12	13	14	15
Behavior scales															
Inattention/ Hyperactivity															
Defiant/Aggressive Behaviors	.64**														
Defiance/Temper	.61**	.92**													
Aggressive Behaviors	.58**	.94**	.73**												
Social Functioning/ Atypical Behaviors	.59**	.58**	.51**	.56**											
Social Functioning	.53**	.52**	.44**	.53*	.93**										
Atypical Behaviors	.53**	.53**	.50**	.49**	.75**	.49**									
Anxiety	.37**	.40**	.40**	.34**	.48**	.35**	.54**								
Mood and Affect	.61**	.70**	.72**	.59**	.72**	.61**	.63**	.68**							
Physical Symptoms	.37**	.35**	.36**	.30**	.39**	.29**	.43**	.67**	.48*						
Developmental Milestone scales															
Adaptive Skills	.32**	.10**	.10*	.09*	.30**	.26**	.23**	.10**	.21**	.13**					
Communication	.38**	.13**	.11**	.13**	.45**	.43**	.29**	.18**	.33**	.17**	.72*				
Motor Skills	.34**	.10**	.11**	.08*	.34**	.31**	.25**	.15**	.26**	.17**	.83**	.78**			
Play	.31**	.11**	.09*	.11**	.45**	.43**	.30**	.15**	.28**	.13**	.63**	.72**	.66**		
Pre-Academic/ Cognitive Skills	.35**	.09*	.08*	.08*	.32**	.31**	.22**	.12**	.25**	.15**	.76**	.86**	.89**	.68**	
Global Development	.38**	.11**	.11**	.11**	40**	.37**	.27**	.16**	.29**	.17**	.87**	.91**	.94**	.77**	.96**

Note. Pair-wise deletion of missing cases was used (N = 663-667).

** = $p < .01$; * = $p < .05$.

Simple structure of EFA of Conners EC^{TM} behavior scales parent version

Item	Factor 1	2	3	4	5	6	7	8
B79. Difficulty focusing[a]	.948							
B72. Short attention span	.928							
B86. Inattentive	.857							
B76. Loses interest	.836							
B90. Fails to finish things	.821							
B34. Difficulty staying in seat	.749							
B12. Not pay attention	.723							
B74. Fidgeting	.702							
B55. "On the go"	.595							
B49. Jumps from one to another	.585							
B81. Restless	.579							
B42. Active when supposed to sit	.522							
B65. Acts before thinking	.470							
B62. Over-stimulated	.345							
B48. Temper outbursts		.873						
B15. Mad easily		.829						
B64. Defiant		.765						
B45. Stubborn		.761						
B 22 Excitable		.609						
B8. Easily frustrated		.570						
B101. Mood changes		.543						
B97. Argues		.507						

B32. Whines/complains	.500			
B11. Eats non-food items	.473			
B99. Refuses to do what he/she is asked to do.	.385			
B92. Bossy	.305			
B91. Picks on others		.680		
B37. Hurt other people’s feelings		.654		
B71. Threatens people		.541		
B106. Gets into fights		.506		
B108. Destroys		.423		
B95. Rude		.406		
B77. Swears		.390		
B73. Play with fire		.372		
B7. Cruel to animals		.332		
B69. Lies/manipulate		.316		
B98. Anxious			.813	
B58. Timid/frightened			.812	
B41. Afraid of specific objects or situations			.518	
B59. Feelings easily hurt			.495	
B66. Afraid to be alone			.485	
B29. Clingy/attached to parent(s)			.370	
B44. Liked by others				.667
B1. Makes friends				.561
B56. Gets along well				.545
B46. Happy for others				.521
B25. Gets invited				.517

B85. Comfort others					.490			
B28. Smiles when others smile					.453			
B21. Affectionate					.367			
B5. Odd/unusual						.497		
B30. Unusual interests						.468		
B61. Rejected (peers)						.445		
B2. Trouble controlling worries						.424		
B50. Repeats body movements						.412		
B36. Says bad things (about self)						.374		
B14. Making repeated sounds						.363		
B47. Driven by a motor						.361		
B51. Picked on by others						.359		
B4. Worries						.343		
B63. Own world						.333		
B38. Anticipates the worst						.332		
B26. Sad or morbid themes in play						.331		
B70. Sad						.309		
B80. Trouble falling asleep							.846	
B24. Trouble falling asleep (alone)							.816	
B33. Wakes up during night and trouble falling back to sleep							.439	
B40. Nightmares/night terrors							.306	
B82. Aches/pains								.764
B20. Stomach aches								.660
B27. Sick (even when nothing is wrong)								.506
Variance in %	21.71	4.51	3.98	3.54	2.04	2.20	1.72	1.50
Eigenvalues	15.41	3.20	2.83	2.52	1.45	1.56	1.22	1.07

Note. [a]For copyright reasons we provided a shortened version of item content for the scales. Please refer to Conners (2009) and Harbarth et al. (2017) for the original items.

S4

Simple structure of EFA of Conners EC^{TM} Developmental Milestone scales parent version

Item	Factor 1	2	3	4	5
DM49. Combine words	.959				
DM70. Complete sentences	.879				
DM34. Words “a,” “an,” and “the”	.867				
DM8. Plural words	.841				
DM31. Verbs (past/future)	.819				
DM25. Explains answers	.696				
DM33. Compares objects	.687				
DM53. Body parts	.607				
DM44. Answers questions about a picture story	.595				
DM38. Labels emotions	.589				
DM17. Uses “I” or “my”	.582				
DM28. Expresses ideas	.574				
DM26. Understands words about time of day	.543				
DM56. Tells name	.538				
DM47. Groups/matches objects	.364				
DM57. Fastens clothing		.841			
DM68. Draws person		.768			
DM75. Cuts neatly		.760			
DM4. Rides bicycle		.725			
DM1. Knows when to go to bathroom		.719			
DM60. Colors within lines		.718			

DM73. Stands on one foot		.715			
DM13. Puts shoes on		.690			
DM21. Draws shapes		.666			
DM24. Climbing stairs		.624			
DM59. Counts at least 10 objects		.591			
DM16. Knows basic shapes		.500			
DM74. Do things that adults do			.846		
DM72. Play different characters			.819		
DM69. Acts out different imaginative stories			.765		
DM58. Uses objects to represent things			.681		
DM62. Understands meaning of gestures				.733	
DM23. Uses gestures				.699	
DM36. One-step directions				.671	
DM3. Communicates wants/needs				.651	
DM46. Clean up toys/belongings				.483	
DM6. Completes household chores				.361	
DM63. Identifies all letters of alphabet					.816
DM5. Says/sings the alphabet					.775
DM10. Ties shoelaces					.593
Variance in %	39.45	6.78	4.56	3.46	2.95
Eigenvalues	15.78	2.79	1.82	1.39	1.18

S5

Simple structure of EFA of Conners EC^{TM} behavior scales childcare-provider version

Item	Factor 1	2	3	4
B8. Difficulty focusing on one thing	.914			
B67. Short attention span	.898			
B85. Inattentive	.885			
B13. Difficulty staying in seat	.796			
B30. Fidgeting	.780			
B39. Jumps from one activity to another	.765			
B33. Loses interest	.752			
B10. Restless/overactive	.729			
B89. Fails to finish things	.693			
B2. Not pay attention	.662			
B91. “On the go”	.651			
B79. Active when supposed to walk/sit.	.626			
B26. Keeps attention on one thing	.622			
B54. Acts before thinking	.590			
B77. Waits	.434			
B36. Perfectionist	.408			
B98. Picks on others		.788		
B108. Manipulative		.764		
B111. Bossy		.697		
B57. Hurt other’s feelings		.651		
B76. Gets into fights		.649		

B105. Argues with adults	.633	
B46. Swears	.633	
B86. Threatens others	.609	
B72. Cold-hearted/cruel	.608	
B102. Destroys things	.604	
B40. Lies/manipulate people	.571	
B20. Temper outbursts	.534	
B73. Rude	.531	
B104. Cruel to animals	.467	
B11. Insists	.460	
B101. Defiant	.421	
B82. Gets upset when making a mistake	.394	
B23. Easily frustrated	.352	
B9. Steals	.346	
B38. Unusual interests	.317	
B7. Sad or morbid themes in play	.310	
B51. Gets invited		.800
B1. Liked by others		.786
B43. Makes friends		.744
B41. Gets along well with others		.699
B52. Happy for others		.599
B31. Ignored by others		.595
B49. Rejected (peers)		.549
B96. Trouble keeping friends		.547
B93. Group activities		.513

B84. Comfort others	.478	
B87. Smiles	.466	
B16. Play alone.	.455	
B5. Not enjoy things	.392	
B103. Not show emotions	.365	
B58. Seeks help	.362	
B22. Odd/unusual	.350	
B61. Eye contact	.341	
B100. Picked on by others	.341	
B74. Anxious		.788
B47. Timid/frightened		.777
B45. Afraid to be alone		.612
B60. Afraid of specific objects or situations		.593
B97. Easily hurt (feelings)		.557
B71. Not like being separated from parents		.539
B4. Anticipates the worst		.521
B99. Cries often/easily		.521
B29. Complains about being sick		.508
B92. Worries		.486
B34. Stomach aches		.453
B65. Nervous/jumpy		.453
B62. Trouble controlling worries		.435
B55. Headaches		.416
B64. Dislikes disruption to routine		.410
B17. Aches/pains		.386

B19. Tired				.315
Variance in %	25.25	6.08	5.14	4.25
Eigenvalues	18.18	4.38	3.70	3.06

S6

Simple structure of EFA of Conners EC^{TM} Developmental Milestone scales childcare-provider version

	Factor					
Item	1	2	3	4	5	6
DM42. Combines words	1.008					
DM58. “a,” “an,” and “the”	.952					
DM12. Complete sentences	.924					
DM18. Answers questions (about picture story)	.873					
DM41. Names opposites	.823					
DM7. Plural words	.788					
DM46. Names things	.773					
DM10. Basic colors	.739					
DM32. Follows directions	.729					
DM57. Compares objects	.726					
DM56. Explains answers to simple problems	.681					
DM1. Understands “more,” “less,” and “same”	.676					
DM48. Body parts	.653					
DM21. Counts from 1-5	.642					
DM43. Expresses ideas	.630					
DM38. Understands words about time	.580					
DM9. Labels emotions	.499					
DM30. Handles small objects	.366					
DM55. Prints first name		1.085				
DM60. Draws a person		.976				
DM15. Points to the correct letter/number		.873				

DM25. Rides bicycle		.831				
DM62. Rhymes words		.749				
DM11. Cuts neatly		.715				
DM49. Draws shapes		.691				
DM27. Colors within the lines		.660				
DM8. Catches a ball (big)		.659				
DM33. Fastens clothing		.637				
DM47. Catches a ball (small)		.461				
DM50. Knows when to go to the bathroom			.985			
DM69. Pulls up pants			.978			
DM52. Fully toilet-trained (day/night)			.744			
DM54. Puts on shirt			.687			
DM44. Puts on jacket			.669			
DM65. Feeds self			.519			
DM13. Hops on one foot			.505			
DM28. Puts shoes on correctly			.436			
DM70. Do things that adults do			.427			
DM37. Drinks (through a straw)				.739		
DM35. Kicks a ball				.665		
DM61. Uses gestures				.551		
DM36. Shows emotions (including facial expressions)					.809	
DM5. Recognizes different emotions					.682	
DM23. Clean up toys/belongings						.773
DM63. Follows one-step directions						.713
Variance in %	48.21	6.07	3.86	3.24	2.52	2.22

Eigenvalues	22.66	2.86	1.81	1.52	1.18	1.05

Table S7

Internal consistency of Conners EC^{TM}

	Parent version						Childcare-provider version					
		age						age				
Scales	Total	2	3	4	5	6	Total	2	3	4	5	6
Behavior scales												
Inattention/Hyperactivity	.94	.89	.95	.95	.94	.93	.95	.94	.94	.94	.96	.94
Defiant/Aggressive Behaviors	.88	.85	.87	.89	.87	.91	.92	.87	.92	.90	.91	.94
Defiance/Temper	.86	.85	.85	.87	.83	.87	.84	.77	.81	.84	.86	.87
Aggressive Behaviors	.76	.55	.68	.79	.78	.86	.88	.88	.89	.83	.87	.93
Social Functioning/Atypical Behaviors	.83	.74	.85	.85	.81	.84	.88	.92	.88	.87	.87	.91
Social Functioning	.78	.67	.80	.80	.78	.77	.84	.87	.86	.84	.84	.87
Atypical Behaviors	.74	.63	.79	.76	.72	.71	.70	.77	.69	.62	.71	.75
Anxiety	.83	.79	.80	.83	.89	.80	.85	.81	.85	.79	.90	.87
Mood and Affect	.76	.76	.76	.75	.78	.76	.75	.70	.67	.56	.83	.82
Physical Symptoms	.68	.60	.64	.67	.74	.62	.68	.61	.58	.49	.77	.78
Sleep Problems	.57	.51	.56	.54	.63	.60	-[a]	-	-	-	-	-
Developmental Milestone scales												
Adaptive Skills	.90	.89	.86	.87	.89	.87	.89	.84	.76	.81	.78	.84
Communication	.91	.90	.88	.91	.93	.89	.93	.89	.93	.87	.89	.93
Motor Skills	.90	.89	.86	.85	.90	.88	.91	.84	.87	.71	.83	.89

Play	.83	.84	.79	.83	.85	.84	.80	.81	.85	.64	.83	.78
Pre-Academic/Cognitive Skills	.93	.93	.89	.90	.92	.90	.95	.84	.91	.90	.90	.93
Global Development	.97	.97	.96	.97	.97	.96	.98	.95	.97	.94	.95	.97

Note. Internal consistency measured with Cronbach's alpha. [a]Childcare-provider rating do not assess "Sleep Problems".

S8

Univariate effects of age on Conners EC^{TM} scales parent version

Scale		Age in years 2[a]	3[b]	4[c]	5[d]	6[e]	*F*(df)	*p*	partial η^2	Pair-wise differences
**Empirical Scales**										
Inattention/ Hyper-activity	M	13.34	13.71	12.37	11.92	12.51	0.91 (4)	.460	-	-
	SE	7.88	10.26	9.47	9.92	9.29				
Defiant/ Aggressive Behaviors	M	12.38	13.69	14.12	11.39	12.26	3.63 (4)	.006	.021	4 > 5
	SE	5.95	7.54	8.08	7.46	7.96				
Social Functioning/ Atypical Behaviors	M	13.17	13.64	14.46	12.93	12.63	1.27 (4)	.281	-	-
	SE	6.03	8.03	8.58	7.02	7.78				
Anxiety	M	9.32	10.17	9.96	8.91	11.06	1.85	.082	-	-
	SE	5.76	5.96	6.62	7.41	6.19				
**Subscales**										
Defiance/ Temper	M	10.90	11.34	11.36	9.34	10.13	3.68 (4)	.006	.021	3, 4 > 5
	SE	5.14	5.88	5.89	5.85	5.56				
Aggressive Behaviors	M	1.49	2.36	2.77	2.05	2.15	4.60 (4)	.001	.026	3, 4 >2
	SE	1.54	2.33	3.01	2.33	3.24				
Social Functioning	M	9.56	9.28	9.88	9.27	8.67	0.89 (4)	.469	-	-
	SE	4.15	5.23	5.60	5.20	4.96				
Atypical Behaviors	M	3.52	4.20	4.34	3.44	3.68	1.92	.106	-	-

	SE	3.06	4.03	4.08	3.27	3.52	(4)			
Sleep Problems	M	2.02	2.05	1.92	1.63	2.20	1.34 (4)	.233	-	-
	SE	1.87	2.02	2.06	2.16	2.20				
**Developmental Milestone scales**										
Global Develop-ment	M	60.07	41.42	27.57	20.76	15.57	65.68 (4)	.001	.275	2 > 3 > 4, 5 > 6
	SE	29.85	22.13	23.04	22.67	17.89				
Adaptive Skills	M	13.43	8.66	5.53	4.01	3.04	61.85 (4)	.001	.263	2 > 3 > 4 > 6
	SE	7.22	5.84	5.48	5.19	4.19				
Communi-cation	M	8.19	4.31	3.38	2.74	1.88	23.62 (4)	.001	.120	2 > 3 > 6
	SE	6.86	4.85	5.27	4.97	3.52				
Motor Skills	M	16.43	12.19	7.91	5.71	4.30	79.44 (4)	.001	.315	2 > 3 > 4 > 5, 6
	SE	7.02	6.12	5.64	5.79	5.00				
Play	M	3.20	1.83	1.58	1.45	1.20	12.55 (4)	.001	.068	2 > 3, 4, 5, 6
	SE	2.89	2.12	2.31	2.12	2.10				
Pre-Academic/ Cognitive Skills	M	18.80	14.42	9.17	6.85	5.18	67.17 (4)	.001	.280	2 > 3 > 4 > 6
	SE	9.41	7.10	7.07	6.96	6.10				

Note. df = degrees of freedom.
[a]n = 105, [b]n = 159, [c]n = 182, [d]n = 169, [e]n = 82.

S9

Univariate effects of sex on Conners EC^{TM} scales parent version

Scale		Sex		F(df)	p	partial η^2
		Males[a]	Females[b]			
**Empirical Scales**						
Inattention/ Hyperactivity	M	13.84	11.65	9.52 (1)	.002	.013
	SE	10.28	8.56			
Defiant/Aggressive Behaviors	M	14.05	11.79	16.30 (1)	.001	.022
	SE	8.19	6.71			
Social Functioning/ Atypical Behaviors	M	14.60	12.37	15.41 (1)	.001	.021
	SE	8.44	6.68			
Anxiety	M	9.65	9.94	0.36 (1)	.551	-
	SE	6.48	6.55			
**Subscales**						
Defiance/Temper	M	11.15	10.22	4.66 (1)	.031	.006
	SE	6.04	5.45			
Aggressive Behaviors	M	2.91	1.59	49.97 (1)	.001	.065
	SE	2.99	1.91			
Social Functioning	M	10.18	8.64	16.30 (1)	.001	.022
	SE	5.56	4.62			
Atypical Behaviors	M	4.20	3.55	5.64 (1)	.018	.008
	SE	4.02	3.27			
Sleep Problems	M	1.89	2.00	0.44 (1)	.508	-
	SE	2.01	2.17			
**Rational Scales**						
Mood and Affect	M	6.79	6.38	1.75 (1)	.186	-
	SE	4.30	3.91			
Physical Symptoms	M	3.74	4.25	3.85 (1)	.050	.005
	SE	3.36	3.61			
**Developmental Milestone scales**						

Global Development	M	36.23	28.43	10.99 (1)	.001	.015
	SE	28.80	24.83			
Adaptive Skills	M	7.65	5.74	15.07 (1)	.001	.021
	SE	6.98	5.91			
Communication	M	4.59	3.30	15.65 (1)	.001	.021
	SE	6.26	4.44			
Motor Skills	M	10.12	8.21	10.20 (1)	.001	.014
	SE	7.36	6.77			
Play	M	2.09	1.51	13.06 (1)	.001	.018
	SE	2.52	2.15			
Pre-Academic/Cognitive Skills	M	11.78	9.68	10.84 (1)	.001	.015
	SE	8.85	8.14			

Note. df = degrees of freedom.
[a]N = 359. [b]N = 358.

S10

Univariate effects of age on Conners EC^{TM} scales childcare provider version

Scale		Age in years 2	3	4	5	6	*F*(df)	*p*	partial η²	Pair-wise differences
**Empirical Scales**										
Inattention/ Hyperactivity	M	16.87	15.17	12.61	14.11	9.91	3.66 (4)	.006	.027	2, 3 > 6
	SE	10.91	11.94	11.09	12.75	11.80				
Defiant/ Aggressive Behaviors	M	6.03	6.06	6.07	7.23	6.94	0.70 (4)	.593	-	-
	SE	6.10	7.39	6.73	7.92	9.04				
Social Functioning	M	17.81	18.83	17.36	17.98	16.75	0.47 (4)	.759	-	-
	SE	11.97	10.47	10.94	10.56	11.44				
Anxiety	M	5.47	5.68	5.29	6.26	5.96	0.67 (4)	.611	-	-
	SE	4.51	5.40	4.42	6.38	6.02				
**Developmental Milestone scales**										
Global Development	M	82.06	48.18	25.28	15.79	10.70	185.67 (4)	.001	.581	2 > 3 > 4 > 5, 6
	SE	21.63	23.23	19.42	14.74	14.61				
Adaptive Skills	M	12.53	4.97	1.68	1.11	0.68	201.22 (4)	.001	.601	2 > 3 >4, 5, 6
	SE	4.95	3.86	2.48	1.99	1.63				
Communication	M	15.31	7.78	4.28	3.04	2.54	69.04 (4)	.001	.340	2 > 3 > 4, 5, 6
	SE	6.34	7.01	5.45	4.12	4.50				
Motor Skills	M	21.48	13.95	7.43	4.05	2.22	206.24	.001	.607	2 > 3 > 4 > 5, 6

	SE	5.23	5.91	5.27	4.00	3.46	(4)			
Play	M	5.34	3.04	1.83	1.52	1.25	40.32 (4)	.001	.232	2 > 3 > 4, 5, 6
	SE	2.57	2.66	2.21	2.16	1.63				
Pre-Academic/ Cognitive Skills	M	27.15	19.73	11.79	7.95	5.97	170.59 (4)	.001	.561	2 > 3 > 4 > 5, 6
	SE	5.29	7.32	6.73	4.88	5.17				

Note. df = degrees of freedom.
[a]n = 62, [b]n = 109, [c]n = 145, [d]n = 155, [e]n = 69.

S11

Univariate effects of sex on Conners EC^{TM} scales childcare provider version

Scale		Sex				
		Males[a]	Females[b]	*F*(df)	*p*	partial η²
**Empirical scales**						
Inattention/Hyperactivity	M	16.03	11.63	18.01	.001	.033
	SE	12.65	11.10	(1)		
Defiant/Aggressive Behaviors	M	7.59	5.62	9.08	.003	.017
	SE	8.60	6.25	(1)		
Social Functioning/Atypical Behaviors	M	20.06	15.89	20.02	.001	.037
	SE	11.74	9.61	(1)		
Anxiety	M	5.50	5.94	0.86	.354	-
	SE	5.58	5.39	(1)		
**Subscales**						
Defiance/Temper	M	4.09	3.66	1.77	.184	-
	SE	4.08	3.44	(1)		
Aggressive Behaviors	M	3.49	1.97	16.62	.001	.031
	SE	5.10	3.32	(1)		
Social Functioning	M	13.73	11.01	19.60	.001	.036
	SE	7.73	6.35	(1)		
Atypical Behaviors	M	3.76	2.85	10.21	.001	.019
	SE	3.78	2.77	(1)		
**Rational Scales**						
Mood and Affect	M	4.70	4.28	1.54	.215	-
	SE	3.95	3.75	(1)		
Physical Symptoms	M	1.79	1.99	0.85	.358	-
	SE	2.57	2.60	(1)		
**Developmental Milestone scales**						
Global Development	M	34.88	27.77	8.12	.005	.015
	SE	30.93	26.34	(1)		

Adaptive Skills	M	3.82	2.80	6.15 (1)	.013	.012
	SE	5.09	4.31			
Communication	M	6.40	4.60	9.90 (1)	.002	.018
	SE	7.08	6.04			
Motor Skills	M	9.43	7.90	5.18 (1)	.023	.010
	SE	8.32	7.16			
Play	M	2.69	1.94	11.37 (1)	.001	.021
	SE	2.77	2.31			
Pre-Academic/Cognitive Skills	M	13.90	12.17	4.95 (1)	.027	.009
	SE	9.37	8.49			

Note. df = degrees of freedom.
[a]N = 257. [b]N = 271.
